# Fermented milk containing *Lactobacillus paracasei* subsp. *paracasei* CNCM I-1518 reduces bacterial translocation in rats treated with carbon tetrachloride

**DOI:** 10.1038/srep45712

**Published:** 2017-04-03

**Authors:** Elisabet Sánchez, Juan C. Nieto, Silvia Vidal, Alba Santiago, Xavier Martinez, Francesc J. Sancho, Pau Sancho-Bru, Beatriz Mirelis, Helena Corominola, Candido Juárez, Chaysavanh Manichanh, Carlos Guarner, German Soriano

**Affiliations:** 1 Department of Gastroenterology, Hospital de la Santa Creu i Sant Pau, Barcelona, Spain; 2 Institut d´Investigacions Biomèdiques (IIB) Sant Pau, Barcelona, Spain; 3 Centro de Investigación Biomédica en Red de Enfermedades Hepáticas y Digestivas (CIBERehd), Instituto de Salud Carlos III, Madrid, Spain; 4 Universitat Autònoma de Barcelona, Bellaterra (Cerdanyola del Vallès), Spain; 5 Department of Immunology, Hospital de la Santa Creu i Sant Pau, Barcelona, Spain; 6 Digestive System Research Unit, Vall d’Hebron Research Institute, Barcelona, Spain; 7 Department of Pathology, Hospital de la Santa Creu i Sant Pau, Barcelona, Spain; 8 Institut Investigacions Biomèdiques August Pi i Sunyer (IDIBAPS), Hospital Clínic de Barcelona, Barcelona, Spain; 9 Department of Microbiology, Hospital de la Santa Creu i Sant Pau, Barcelona, Spain; 10 Danone Nutricia Research, Barcelona, Spain

## Abstract

Probiotics can prevent pathological bacterial translocation by modulating intestinal microbiota and improving the gut barrier. The aim was to evaluate the effect of a fermented milk containing *Lactobacillus paracasei* subsp*. paracasei* CNCM I-1518 on bacterial translocation in rats with carbon tetrachloride (CCl_4_)-induced cirrhosis. Sprague-Dawley rats treated with CCl_4_ were randomized into a probiotic group that received fermented milk containing *Lactobacillus paracasei* subsp. *paracasei* CNCM I-1518 in drinking water or a water group that received water only. Laparotomy was performed one week after ascites development. We evaluated bacterial translocation, intestinal microbiota, the intestinal barrier and cytokines in mesenteric lymph nodes and serum. Bacterial translocation decreased and gut dysbiosis improved in the probiotic group compared to the water group. The ileal β-defensin-1 concentration was higher and ileal malondialdehyde levels were lower in the probiotic group than in water group. There were no differences between groups in serum cytokines but TNF-α levels in mesenteric lymph nodes were lower in the probiotic group than in the water group. Fermented milk containing *Lactobacillus paracasei* subsp*. paracasei* CNCM I-1518 decreases bacterial translocation, gut dysbiosis and ileal oxidative damage and increases ileal β-defensin-1 expression in rats treated with CCl_4_, suggesting an improvement in the intestinal barrier integrity.

Bacterial translocation of enteric bacteria to mesenteric lymph nodes or other extra intestinal sites can cause severe infections^1^,^2^,^3^. However, the interplay between these bacteria and/or their products and the host immune system can also contribute to the development of a proinflammatory state^1^,^2^,^3^. Inflammation is in turn related to multi-organ failure in critically ill patients^4^, to metabolic diseases and their consequences^5^, and to disease progression and development of complications in patients with liver diseases^1^,^2^,^3^.

The mechanisms proposed to explain pathological bacterial translocation include alterations in gut microbiota, impaired intestinal barrier, and altered immune defenses^1^,^2^,^3^. Possible methods to prevent this phenomenon have been largely studied in experimental models of liver disease in rodents^6^. The administration of carbon tetrachloride (CCl_4_) is one of the most widely used experimental models of liver disease and cirrhosis^7^,^8^,^9^,^10^. Several studies using this experimental model have demonstrated the high efficacy of antibiotics to decrease intestinal bacterial overgrowth and prevent bacterial translocation^11^,^12^,^13^. However, antibiotics favour the development of bacterial resistance, mainly if they are administered for long periods of time^14^. Therefore, alternative treatments have been proposed, including beta-blockers^15^, bile acids^16^, prokinetics^17^, antioxidants^18^ and probiotics^10^,^13^,^18^.

Probiotics are living organisms that produce a beneficial effect to the host when administered in a sufficient amount^19^. They can prevent bacterial translocation by decreasing intestinal bacterial overgrowth and improving intestinal barrier and immune disturbances^20^,^21^. However, previous data on the prevention of bacterial translocation with probiotics in rodents treated with CCl_4_ are contradictory. Although *Lactobacillus* GG^13^ and *Lactobacillus johnsonii* La1^22^ failed to show a benefit in bacterial translocation, other studies observed a reduction in bacterial translocation and proinflammatory state after treatment with *Bifidobacterium pseudocatenulatum* CECT7765 in Balb/c mice^10^ and a multispecies probiotic combination in Sprague-Dawley rats^9^. These favourable results seem to be a consequence of an improvement in the intestinal barrier^9^,^10^.

*Lactobacillus paracasei* subsp*. paracasei* CNCM I-1518 has been shown to improve the gut barrier and to reduce pro-inflammatory cytokines in Peyer’s patches and bacterial translocation in experimental colitis^23^,^24^. However, this probiotic has not yet been evaluated in experimental cirrhosis. Actimel^®^ (Danone, Palaiseau, Cedex, France) is a commercial dairy product widely used in humans that contains fermented milk with *L. paracasei* subsp. *paracasei* CNCM I-1518, in addition to the yogurt bacteria *Streptococcus thermophilus* and *L. bulgaricus*, and vitamins B_6_ and D.

The aim of the present study was to compare the effects of fermented milk containing *L. paracasei* subsp. *paracasei* CNCM I-1518 (Actimel^®^) with water on bacterial translocation, gut microbiota, intestinal barrier and inflammatory response in the experimental model of rats treated with CCl_4_.

## Results

Fifty-four rats were included in the study. Forty-four rats were treated with CCl_4_ and 5 died before week 6. At week 6 of treatment with CCl_4_, the remaining thirty-nine rats were randomized into the probiotic group (*n* = 20) or the water group (*n* = 19). The remaining 10 rats made up the control group.

The time elapsed between randomization and the study end (death or laparotomy, performed 1 week after rats developed ascites) was similar in the probiotic group and in the water group (5.1 ± 0.6 vs 5.8 ± 0.8 weeks, *P* = 0.60). When considering only the rats that reached laparotomy, the time elapsed was 6.3 ± 0.7 and 6.1 ± 0.8 weeks, respectively (*P* = 1).

During the study, the total dose of CCl_4_ received per rat was similar in the probiotic group and in the water group: 2873.0 ± 357.2 vs 2986.3 ± 447.4 μL (*P* = 0.94).

The mean daily volume of water with probiotic treatment that the rats in the probiotic group drank was 57.8 ± 3.3 mL/day. The mean daily volume of water that the rats in the water group drank was 27.1 ± 1.3 mL/day (*P* < 0.001 with respect to probiotic group). The mean daily volume of water that the rats in the control group drank was 34.6 ± 0.8 mL/day (*P* < 0.001 with respect to the probiotic group and *P* = 0.001 with respect to the water group).

The weight of chow that was eaten by the rats during the study was 21.3 ± 0.9 g/day in the probiotic group, 25.9 ± 1.2 g/day in the water group (*P* = 0.01 with respect to probiotic group) and 29.5 ± 1.3 g/day in the control group (*P* < 0.001 with respect to probiotic group, *P* = 0.08 with respect to water group). Therefore, the kcals received with the chow were lower in the probiotic group (61.8 ± 2.8 kcal/day) than in the water group (75.1 ± 3.6, *P* = 0.01) and the control group (85.5 ± 3.9, *P* < 0.001, *P* = 0.08 with respect to water group). As the rats in the probiotic group received a supplementary 20.5 ± 1.2 kcal/day with the fermented milk diluted in water, the total kcals received by the rats did not differ between the three groups (82.4 ± 3.3 in the probiotic group, 75.1 ± 3.6 in the water group, and 85.5 ± 3.9 in the control group, *P* NS). The weight of the rats at laparotomy was also similar in the two groups treated with CCl_4_ (353.3 ± 30.9 g in the probiotic group and 388.1 ± 11.5 g in the water group, *p* = 0.39).

The estimated mean daily dose of bacteria received by rats in the probiotic group was 2.9 ± 0.1 × 10^9^ colony forming units (cfu).

### Probiotic treatment did not reduce mortality or ascites formation

The mortality rate during the study was 35% (7/20) in the probiotic group, 31% (6/19) in the water group (*P* = 0.8 with respect to probiotic group), and 0% (0/10) in the control group (*P* = 0.06 with respect to the other two groups). The number of rats that developed ascites demonstrated by paracentesis was 80% (16/20) in the probiotic group and 79% (15/19) in the water group (*P* = 0.9). The cumulative probability of developing ascites at week 18 was 88% in the probiotic group and 86% in the water group (*P* = 0.52).

### Probiotic treatment reduced bacterial translocation

**Figure 1:**
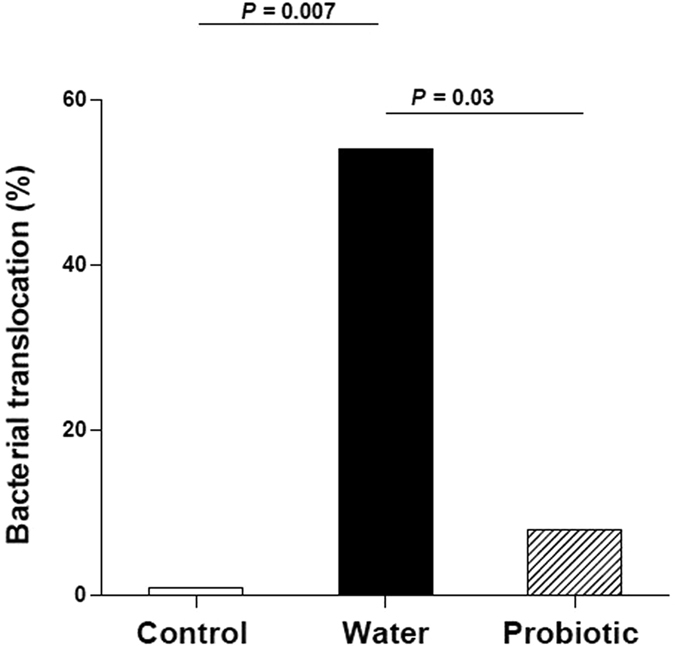
Incidence of bacterial translocation. Control rats (control group) (*n* = 10), rats treated with CCl_4_ and water (water group) (*n* = 19) and rats treated with CCl_4_ and fermented milk containing *Lactobacillus paracasei* ssp *paracasei* CNCM I-1518 (probiotic group) (*n* = 20). CCl_4_, carbon tetrachloride.

Figure 1 shows the incidence of bacterial translocation was significantly lower in the probiotic group (7.7%, 1/13) than in the water group (54%, 7/13) (*P* = 0.03). The incidence of bacterial translocation was higher in the water group than in control rats (0%, 0/10) (*P* = 0.007). Table 1 shows the bacteria and the sites where bacteria were isolated. *Escherichia coli* and *Enterococcus* spp. were the most frequently detected bacteria in cirrhotic rats, mainly in the mesenteric lymph nodes.

### Effect of probiotic treatment on intestinal microbiota

**Figure 2:**
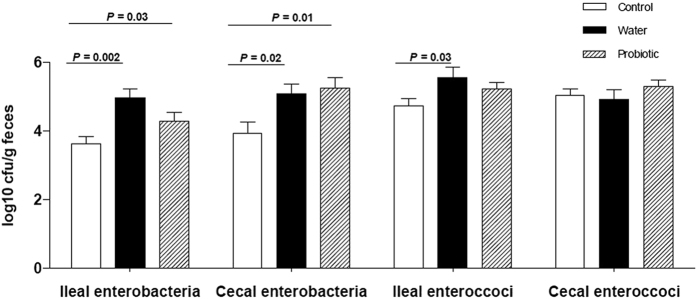
Bacterial concentration in ileal and cecal content. Control rats (control group) (*n* = 10), rats treated with CCl_4_ and water (water group) (*n* = 19) and rats treated with CCl_4_ and fermented milk containing *Lactobacillus paracasei* ssp. *paracasei* CNCM I-1518 (probiotic group) (*n* = 20). Values are mean ± SEM. cfu, colony forming units. CCl_4_, carbon tetrachloride.

Figure 2 shows the concentration of enterobacteria and enterococci in the ileal and cecal contents in the three study groups analysed by microbial cultures. The two groups of rats treated with CCl_4_ showed higher bacterial counts than control rats. This difference was statistically significant for ileal (*P* = 0.03) and cecal enterobacteria (*P* = 0.01) in the probiotic group, and for ileal enterobacteria (*P* = 0.002) and enterococci (*P* = 0.03) and cecal enterobacteria (*P* = 0.02) in the water group. However, we did not observe differences between the probiotic group and the water group in any of the microbiological determinations.

**Figure 3:**
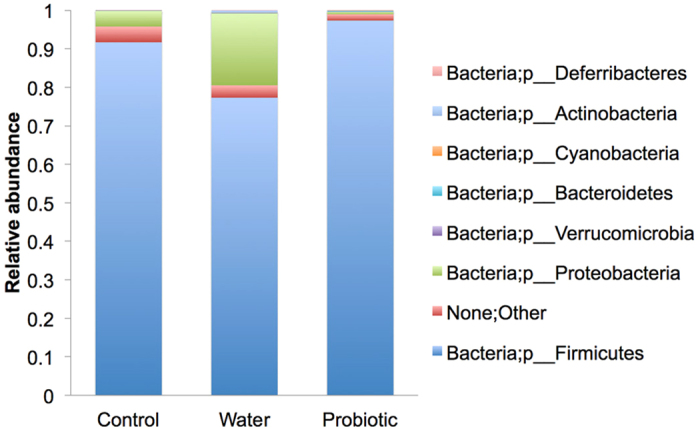
Relative abundance of the microbiome community at the phylum level. Firmicutes was shown to be significantly different between the water group compared to the control and the probiotic groups (*P* = 0.01; FDR = 0.07).

However, the microbiome evaluation of ileal content by 16S rRNA analysis showed at the phylum level that the relative abundance of Firmicutes was significantly lower in the water group than in the control and probiotic groups (*P* = 0.01; FDR = 0.07) (Fig. 3). Moreover, there was a non-significant trend to a higher abundance of Proteobacteria in the control and the probiotic groups than in the water group.

### Probiotic treatment reduced tumor necrosis factor-alpha (TNF-α) in mesenteric lymph nodes but did not change serum cytokines

**Figure 4:**
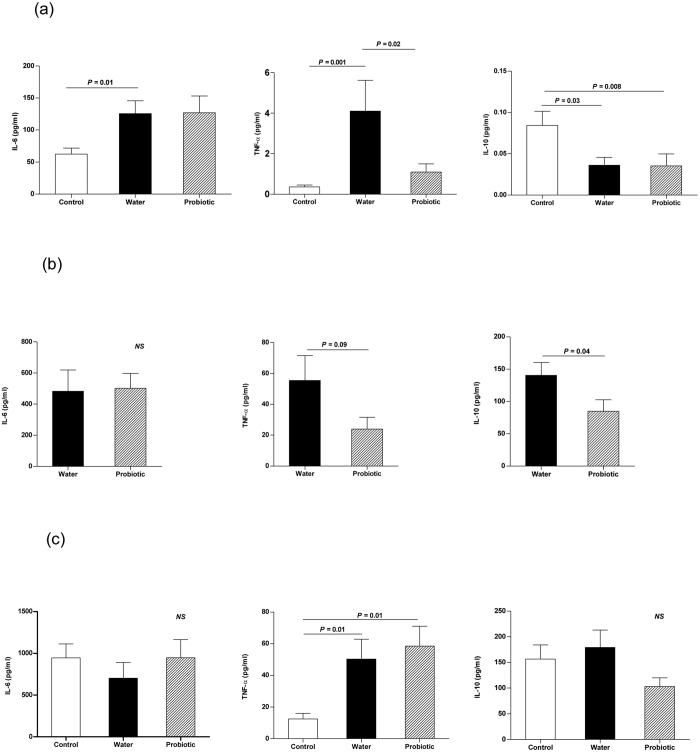
Inflammatory response. Control rats (control group) (*n* = 10), rats treated with CCl_4_ and water (water group) (*n* = 19) and rats treated with CCl_4_ and fermented milk containing *Lactobacillus paracasei* ssp. *paracasei* CNCM I-1518 (probiotic group) (*n* = 20). (**a**) Mesenteric lymph nodes, (**b**) ascitic fluid, and (**c**) serum. Values are mean ± SEM. CCl_4_, carbon tetrachloride, IL-6, interleukin-6; TNF-α, tumor necrosis factor-alpha; IL-10, interleukin-10.

Figure 4a shows cytokine concentrations in mesenteric lymph nodes. Interleukin-6 (IL-6) (*P* = 0.01) and TNF-α (*P* = 0.001) were higher and interleukin-10 (IL-10) (*P* = 0.03) was lower in the water group than in the control group, and TNF-α was lower in the probiotic group than in the water group (*P* = 0.02). Figure 4b shows ascitic fluid cytokine concentrations. There was a trend for a lower TNF-α (*P* = 0.09) and statistically significant lower IL-10 levels (*P* = 0.04) in the probiotic group than in the water group. Figure 4c shows serum cytokine concentrations. TNF-α levels were higher in the two groups treated with CCl_4_ than in the control group (*P* = 0.01 with respect to the probiotic group and to the water group). No differences in serum cytokine concentrations were found between the water and the probiotic groups.

### Probiotic treatment did not modify body weight and spleen/rat weight ratio

Body weight at laparotomy was higher in the control group (484.2 ± 20.6 g) than in the two groups of CCl_4_ treated rats (*P* < 0.001), but it was similar in the probiotic group (353.3 ± 30.9 g) and in the water group (388.1 ± 11.5 g) (*P* = 0.39). The spleen/body weight ratio was lower in control rats (0.0026 ± 0.0001) than in the two groups of rats with cirrhosis (*P* = 0.001), and it was similar in the probiotic group (0.0059 ± 0.0006) and in the water group (0.0066 ± 0.0003) (*P* = 0.45).

### Probiotic treatment increased β-defensin-1 and decreased malondialdehyde (MDA) in ileal samples

**Figure 5:**
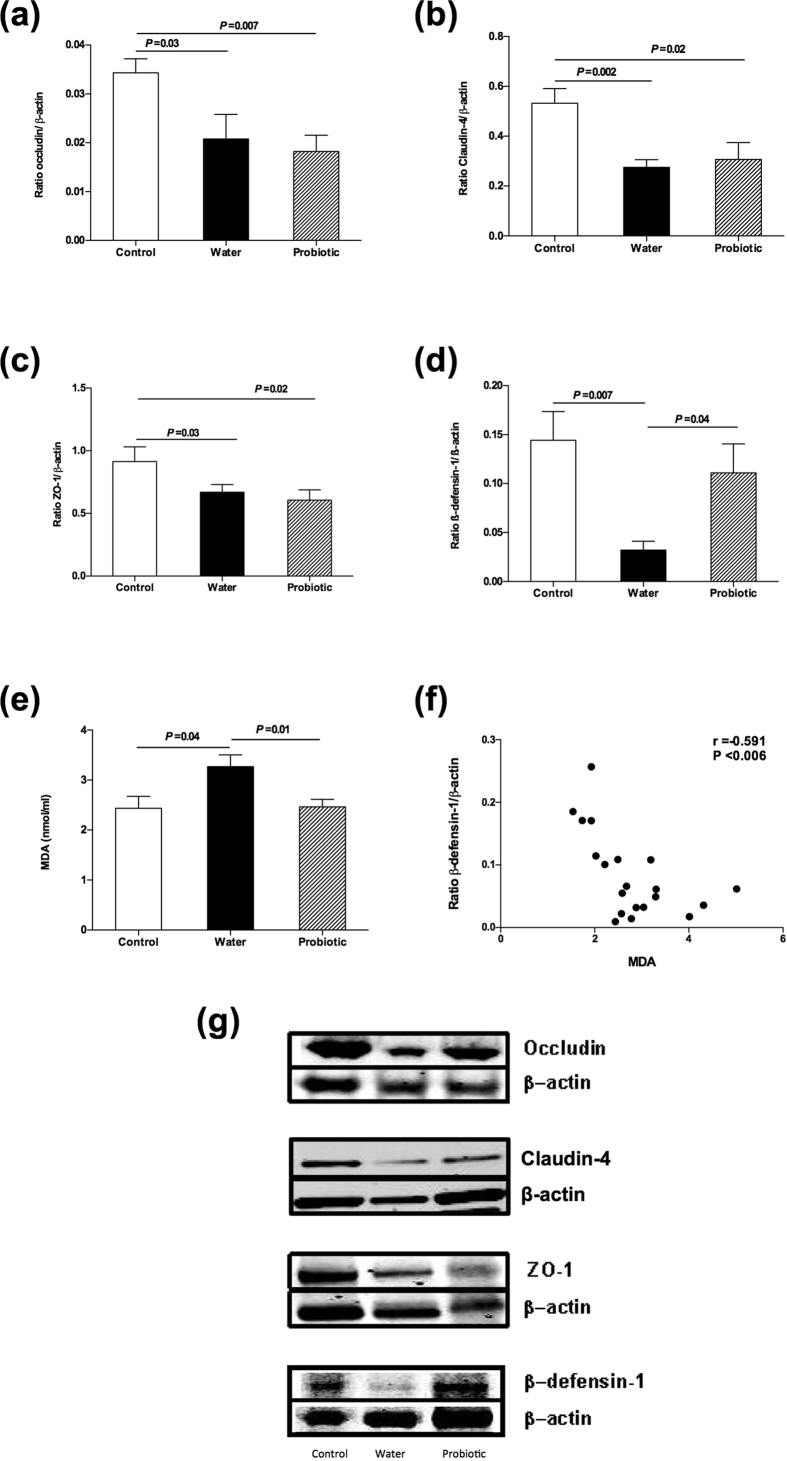
Intestinal barrier. Control rats (control group) (*n* = 10), rats treated with CCl_4_ and water (water group) (*n* = 19) and rats treated with CCl_4_ and fermented milk containing *Lactobacillus paracasei* ssp. *paracasei* CNCM I-1518 (probiotic group) (*n* = 20). Western blot of ileal samples for (**a**) occludin, (**b**) claudin-4, (**c**) zonula occludens (ZO)-1 and (**d**) β-defensin-1, respectively, (**e**) ileal MDA levels, (**f**) correlation between ileal expression of β-defensin-1 and MDA, and (**g**) representative image of western blot of occludin, claudin-4, ZO-1 and β-defensin-1 in control, water or probiotic group. Values are mean ± SEM. CCl_4_, carbon tetrachloride, MDA, malondialdehyde. Cropped membranes are displayed and full-length blots are included in Supplementary Information.

Figure 5 shows the expression of ileal occludin, claudin-4, zonula occludens-1 and β-defensin-1 were lower in the water group than in control rats. Ileal MDA levels were higher in the water group than in control group. Rats receiving probiotic treatment showed an increase in ileal β-defensin-1 and a decrease in ileal MDA with respect to the water group (*P* = 0.04 and *P* = 0.01, respectively). There were no statistical differences between probiotic and water groups regarding ileal occludin, claudin-4 and zonula occludens-1. Considering all the rats treated with CCl_4_, ileal β-defensin-1 expression was lower in rats with bacterial translocation than in rats without (ratio 0.017 ± 0.004 vs 0.064 ± 0.018, *P* = 0.04). Moreover, we found a negative correlation between ileal β-defensin-1 and MDA (r = −0.59, *P* = 0.006).

### Probiotic treatment did not modify liver damage

**Figure 6:**
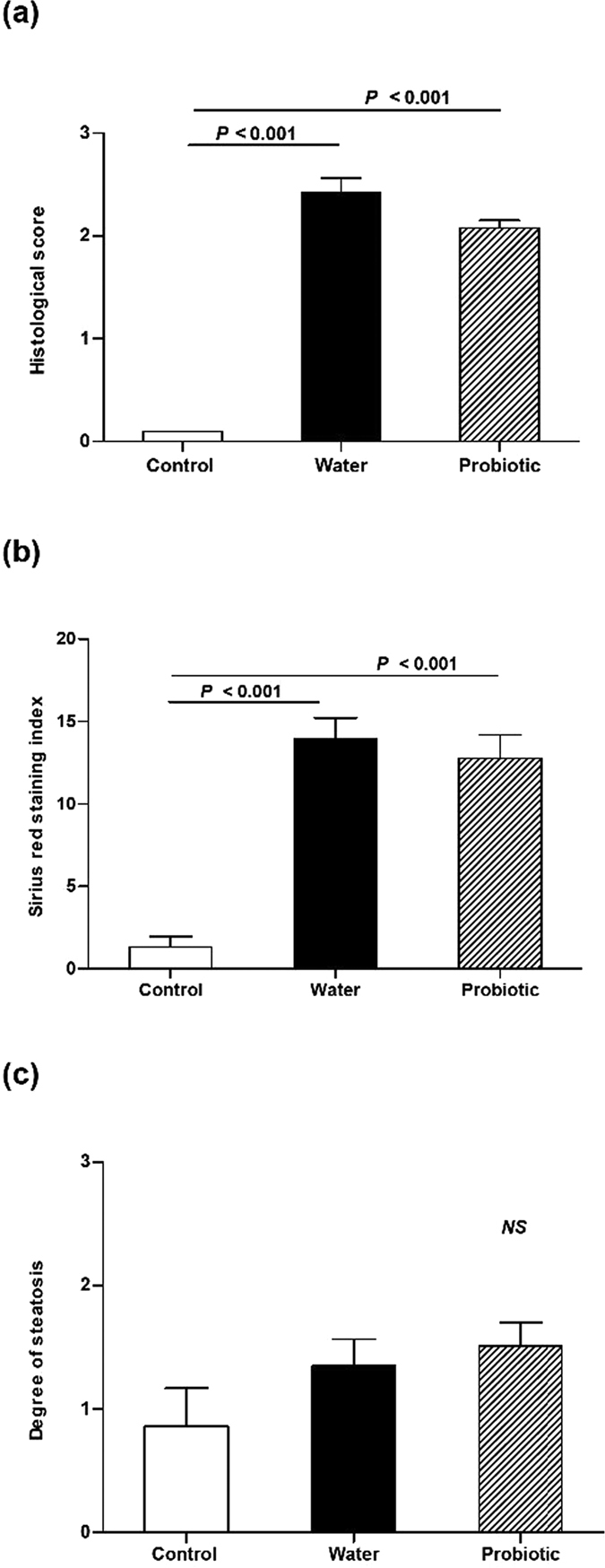
Liver damage. Control rats (control group) (*n* = 10), rats treated with CCl_4_ and water (water group) (*n* = 19) and rats treated with CCl_4_ and fermented milk containing *Lactobacillus paracasei* ssp. *paracasei* CNCM I-1518 (probiotic group) (*n* = 20). (**a**) Histological score, (**b**) Sirius red staining, and (**c**) degree of steatosis. Values are mean ± SEM. CCl_4_, carbon tetrachloride.

Figure 6 shows the degree of liver damage assessed by the histological score, Sirius red staining and the degree of steatosis. The two groups of CCl_4_ treated rats had higher histological scores and a higher percentage of Sirius red staining than control rats. However, we did not find differences in these parameters between the probiotic group and the water group. We did not observe statistical differences between the three groups in the degree of steatosis.

## Discussion

The main finding in this study was that probiotic treatment with fermented milk containing *Lactobacillus paracasei* subsp. *paracasei* CNCM I-1518 decreased bacterial translocation in rats treated with CCl_4_.

Previous experimental studies evaluating probiotics in rodents treated with CCl_4_ reported contradictory results. Two studies failed to show an effect of *Lactobacillus* GG^13^ or *Lactobacillus johnsonii* La1^22^ on bacterial translocation. However, Moratalla *et al*.^10^ observed a decrease in bacterial DNA translocation in animals treated with *Bifidobacterium pseudocatenulatum* CECT7765 in an experimental model of Balb/c mice with CCl_4_-induced cirrhosis submitted to an oral overload of *E. coli*. Moreover, our group recently reported a decrease in bacterial translocation in CCl_4_-treated rats receiving a multispecies probiotic mixture^9^.

These contradictory findings could be due to several factors. First, different probiotics may produce different effects in a given experimental or clinical situation^16^,^17^,^18^. Second, differences among studies in the experimental model and in the duration of probiotic treatment^9^,^10^,^13^,^22^ could explain some contradictory results. For the present study, we chose *L. paracasei* subsp*. paracasei* CNCM I-1518 because treatment with this probiotic has previously shown to reduce bacterial translocation in rats with colitis induced by the instillation of trinitrobenzene sulphonic acid^24^. With respect to the treatment schedule, we started probiotic administration at week 6 of CCl_4_ administration and the rats received the probiotic until one week after development of ascites, for a mean of 6 weeks. We used this schedule because it is similar to that used in our previous study that showed a decrease in bacterial translocation with a different probiotic treatment^9^.

The decrease in bacterial translocation observed in the probiotic group in the present study could be due to several non-excluding mechanisms: a decrease in intestinal bacterial overgrowth, an improvement in the intestinal barrier, or modulation of immune response^1^,^2^,^3^. Using microbiological culture methods, we found the intestinal concentration of the bacteria most frequently translocated in our study (enterobacteria and enterococci) was similar in the probiotic group and in the water group. However, the 16S rRNA analysis of the ileal content showed a significant increase in the relative abundance of Firmicutes and a non-significant trend to a decrease in Proteobacteria in the probiotic group in comparison with the water group. As cirrhotic rats from the water group presented a decrease in Firmicutes and a trend to an increase in Proteobacteria with respect to control healthy rats, our findings suggest that the reduction in bacterial translocation that we observed in the probiotic group is partially due to an improvement in the gut dysbiosis.

Dysfunction of the intestinal barrier is considered a relevant mechanism to explain pathological bacterial translocation^1^,^2^,^3^. Tight junctions and antimicrobial peptides are important components of the gut barrier, and alterations in these components may contribute to bacterial translocation^1^,^25^,^26^. Few data, however, are available in cirrhosis^1^,^27^,^28^. In CCl_4_-induced cirrhotic rats, Teltschik *et al*.^26^ observed a relationship between bacterial translocation and deficiencies in the intestinal expression of Paneth cell α-defensins, mainly cryptidin 5 and 7, together with elevated or unchanged non-Paneth cell β-defensins expression. In the present study, we observed a decrease in the ileal expression of tight junction proteins, such as claudin-4, occludin and zonula occludens-1, and the antimicrobial peptide β-defensin-1 in the water group in comparison with control rats. Similar findings have been previously described by our group^9^. Probiotic treatment restored the decreased β-defensin-1 expression without significant changes in tight junction proteins. Other authors have also reported an association between the decrease in bacterial translocation and the increase in the expression of intestinal antimicrobial peptides, including β-defensin-1, in another experimental model, in mice submitted to repeated restraint stress undergoing moderate exercise^29^. In an experimental model of dextran sulphate sodium-induced colitis in Balb/c mice, *L. paracasei* subsp. *paracasei* CNCM I-1518 has been observed to improve the intestinal barrier, the gut permeability and the expression of zonula ocludens-1^23^.

Moreover, in the present study we found a decrease in the oxidative damage in the ileum of rats from the probiotic group. Intestinal oxidative damage has been associated with impairment in the intestinal barrier, delayed intestinal transit time^30^,^31^, and bacterial translocation in rats treated with CCl_4_^32^. Our findings suggest that the increase in β-defensin-1 expression and the decrease in oxidative damage in the gut barrier are relevant for explaining the reduction in bacterial translocation observed in the group treated with the probiotic.

Interestingly, in a previous study using the same experimental model, rats treated with a different probiotic mixture showed a decrease in bacterial translocation and an increase in occludin ileal expression, without changes in β-defensin-1^9^. These data suggest that different probiotics could have the same effect (in this case, a decrease in bacterial translocation) via different mechanisms.

Pathological bacterial translocation has been related to the proinflammatory state. In our study, rats treated with the probiotic presented a decrease in the levels of TNF-α in mesenteric lymph nodes. Other authors have also observed in an experimental model of dextran sulphate sodium-induced colitis in mice, that *L. paracasei* subsp. *paracasei* CNCM I-1518 reduced the production of pro-inflammatory cytokines in Peyer’s patches^23^. This immune modulation could be due to the interaction between probiotic bacteria and the immune system^33^,^34^ in mesenteric lymph nodes and/or to the decrease of bacterial translocation to the mesenteric lymph nodes by potentially pathogenic bacteria, such as enterobacteria and enterococci^1^,^2^,^3^. However, these changes in the mesenteric lymph nodes in the probiotic group were not associated with differences in the serum cytokine profile. Probably as a consequence of this lack of systemic effect, we did not observe significant variations either in the spleen/body weight ratio as a surrogate marker of portal pressure and hemodynamic disturbances, or in the incidence of ascites in the probiotic group with respect to the water group. In a previous study using the same experimental model, we observed that treatment with a different multispecies probiotic was accompanied by a decrease in serum TNF-α, spleen/body weight ratio and ascites formation^9^.

Bacterial translocation and a proinflammatory state are relevant for promoting liver damage^1^,^3^,^35^. In our study, however, in spite of the reduction in bacterial translocation observed in the probiotic group, we did not find any significant effect on liver damage as assessed by the histological score and collagen deposition. This finding can be related to the absence of an effect of the probiotic treatment on the systemic inflammatory profile.

This study has a major limitation. We wanted to evaluate a commercial dairy product that contained not only *L. paracasei* subsp. *paracasei* CNCM I-1518 but also the yogurt bacteria *S. thermophilus* and *L. bulgaricus*, components resulting from milk fermentation, vitamins B_6_ and D, and the milk itself. We can not therefore be sure if the effects observed in the probiotic group are due to *L. paracasei* subsp. *paracasei* CNCM I-1518 or to any other components of the mixture. It has been suggested that supplementation with vitamin D can protect against bacterial infections due to its immunomodulatory effects^36^. However, when evaluating the effects of a fermented product it is difficult to appraise the relative contribution of the different components, some of which are generated during the fermentation process^37^. Moreover, we are aware that we induced a significant nutritional change in the rats in the probiotic group. These rats received double the oral volume of fluid than the rats from the water group received, and although the mean total daily kcals were similar, the composition of the diet in the probiotic group was clearly different from that in the water group. This could affect the results, as the impact of the diet on the gut microbiota and bacterial translocation is well known^5^. Finally, to avoid the risks and interference with results that daily instrumentation could produce in such a relatively long-term study, we did not administer the probiotic by gavage but ad libitum in drinking water. This could make it difficult to know the exact dose of probiotic received by the rats. However, we recorded the total daily volume of the probiotic treatment that the rats drank and the calculated mean daily amount of bacteria administered was in the range of the scheduled dose.

In conclusion, our findings show that fermented milk containing *Lactobacillus paracasei* subsp. *paracasei* CNCM I-1518 decreases bacterial translocation, gut dysbiosis and ileal oxidative damage and increases ileal β-defensin-1 expression in rats treated with CCl_4_.

## Methods

### Animals

We included Male Sprague-Dawley rats (Harlan Laboratories). They were individually caged and exposed to a 12:12 light/dark cycle and were on a constant room temperature of 21 °C. They were allowed free access to rat chow (A04, SAFE, Augy, France) and drinking water. This study was approved by the Animal Research Committee at the Institut de Recerca of Hospital de la Santa Creu i Sant Pau (Barcelona, Spain) and by the Departament d’Agricultura, Ganaderia, Pesca, Alimentació i Medi Natural (DAAM) de la Generalitat de Catalunya. Animals received human care and all methods were performed in accordance with the relevant guidelines and regulations according to the criteria outlined in the Guide for the Care and Use of Laboratory Animals^38^.

### CCl_4_ administration

We administered CCl_4_ following Runyon *et al*’s method^39^ to induce cirrhosis. Rats weighing 100–120 g were fed standard rodent chow (A04, SAFE, Augy, France) and treated with 1.5 mM/L phenobarbital (Luminal, Kern Pharma S.L., Barcelona, Spain) in drinking water to potentiate the effect of CCl_4_. When they reached a weight of 200 g, weekly doses of CCl_4_ (Sigma-Aldrich Química S.L., Tres Cantos, Madrid, Spain) were administered into the stomach using a stainless steel feeding tube (Popper and Sons, New Hyde Park, NY, USA) and a sterile pyrogen-free syringe (KD Medical GMBH Hospital, Berlin, Germany), without anesthesia. The initial dose of CCl_4_ was 20 μL and subsequent doses were calculated according to the weight changes at 48 hours after the last dose, as previously reported^8^.

### Experimental design

At 6 weeks, the rats under induction of cirrhosis by the administration of CCl_4_ were randomized to receive milk fermented by *Lactobacillus paracasei* subsp. *paracasei* CNCM I-1518 in drinking water (probiotic group) or drinking water alone (water group) until laparotomy. Another group of healthy rats without induction of cirrhosis receiving drinking water (control group) was also included. All the rats from the three groups were allowed free access to rat chow and water with or without the probiotic (probiotic group, and water and control groups, respectively). A group receiving non-fermented milk was not included in the present study because we aimed to compare probiotic administration with the standard care, and milk is not included in the standard care of the rats in this experimental model. Mortality and the development of ascites during the study were recorded. In rats receiving CCl_4_, laparotomy was performed one week after the development of ascites confirmed by paracentesis. The moment to perform the laparotomy in control rats was decided by matching with rats receiving CCl_4_.

### Administration of *L. paracasei* subsp. *paracasei* CNCM I-1518

The fermented milk (Actimel^®^, Danone, Palaiseau, Cedex, France) contained the probiotic strain *Lactobacillus paracasei* subsp. paracasei CNCM I-1518 combined with two bacteria commonly used as yogurt starters, *Streptococcus thermophilus* and *Lactobacillus bulgaricus*. The microbial concentration of the fermented milk at the end of shelf-life met the target of 1 × 10^8^ cfu/g of *L. paracasei* subsp. *paracasei* CNCM I-1518. *S. thermophilus*, and *L. bulgaricus* were also present in the final product at levels >10^7^ cfu/g. The product used in this study also contained 0.42 mg of Vitamin B6 and 0.75 μg of vitamin D per 100 mL.

The product was diluted 1:1 with drinking water to achieve a final bacterial concentration of 0.5 × 10^8^ cfu/mL. The doses were prepared in 100 mL containers every 12 hours during all the study. This schedule was chosen taking into account the mean daily volume of water that rats usually drink, in order to achieve the administration of a daily dose of probiotic of 1–5 × 10^9^ cfu, previously used with other probiotics in experimental studies^9^,^13^,^18^. Rats drank ad libitum and the total volume of mixture they drank every 12 hours was recorded to check the dose of probiotic administered to rats. We also recorded daily the volume of probiotic mixture or water that the rats drank, the weight of chow they ate, the total kcals received, and the proportion of kcals corresponding to chow and to fermented milk.

### Laparotomy

On the last day of the study, we performed a laparotomy under anesthesia with 10 mg/kg of xylacine (Rompun, Bayer, Leverkusen, Germany) and 50 mg/kg of ketamine (Ketolar, Parke-Dawis, New York, NY, USA) under sterile conditions. Samples of ascitic fluid, mesenteric lymph nodes, blood, liver, spleen, ileal stools and terminal ileal wall, cecal stools, and pleural fluid were collected in this sequence. Rats were euthanized using intravenous sodium thiopentate (Penthotal, Abbott Laboratories, Chicago, IL, USA). Blood samples were collected in BD Vacutainer tubes (BDbiosciences, San Jose, CA, USA) containing EDTA and centrifuged. The supernatants were recollected and frozen at −80 °C for later analysis. The other samples were stored at −80 °C. The spleen/body weight ratio was calculated and used as surrogate marker of portal hypertension^32^,^40^.

### Bacterial cultures

Samples of homogenized mesenteric lymph nodes, ascitic fluid, pleural fluid, spleen and liver were inoculated on Columbia blood agar, Columbia CNA agar, and the chromogenic media CPS ID3 (BioMérieux, Marcy l’Étoile, France) and incubated for 48 h at 37 °C in an aerobic atmosphere. Isolates were presumptively identified according to their growth and morphology. Bacterial translocation was considered as the presence of a positive culture in mesenteric lymph nodes, liver, spleen, or ascitic or pleural fluids^18^.

During laparotomy, samples of cecal and ileal content weighing 0.2 g were collected, homogenized and diluted with 2 mL of normal saline in sterile conditions. The gut bacterial concentration was quantified by performing serial decimal dilutions. Samples of 100 μL of each dilution were inoculated on Columbia blood agar, Columbia CNA agar, and the chromogenic media CPS ID3 (BioMérieux, Marcy l’Étoile, France). After incubation for 48 h at 37 °C in an aerobic atmosphere, the colonies were counted. Counts are expressed as log_10_ cfu/g of fresh fecal sample with a detection limit at 10^3^ cfu/g^18^.

### Microbiome analysis

We analyzed the microbiome of ileal content from 33 rats (23 cirrhotic rats, n = 13 treated with the probiotic and n = 10 treated with water; and 10 control rats). All the samples were subjected to genomic DNA extraction using a previously described method^41^. To chemically lyse the samples, we added 250 μl of 4 M guanidine thiocyanate, 40 μl of 10% N-lauroylsarcosine and 500 μl of 5% N-lauroylsarcosine before incubation at 70 °C. Further mechanical disruption was carried out using a Mini-Beadbeater-16 (Biospec Products©) to extract the DNA. To clear lysates, enzymatic digestion of RNA was performed by additing of 2 μl of a 10-mg/ml solution of RNAase, and the resulting DNA was precipitated and further ethanol-purified. Pure DNA was re-suspended in 200 μl Tris-EDTA buffer.

To analyse bacterial composition, we subjected extracted genomic DNA to PCR-amplification of the V4 hypervariable region of the 16S rRNA gene as previously described^41^. Amplicons were purified using the QIAquick PCR Purification Kit (Qiagen, Barcelona, Spain) according to the manufacturer’s instructions, and further quantified using a NanoDrop ND-1000 Spectrophotometer (Nucliber©). The purified amplicons were pooled in equal concentration and finally subjected to sequencing using the Illumina Miseq platform at the Autonomous University of Barcelona (UAB, Spain).

For microbiome analysis, we loaded the raw sequences into the QIIME 1.9.1 pipeline and performed the quality filtering analysis as previously described^42^. After filtering, from 33 fecal samples, we obtained a total of 496761 of high-quality sequences with a number of reads ranging from 1435 to 40735 per sample. We used the USEARCH^43^ algorithm to cluster similar filtered sequences into Operational Taxonomic Units (OTUs) based on a 97% similarity threshold. We then identified and removed chimeric sequences using UCHIME^44^. Since each OTU can comprise many related sequences, we picked a representative sequence from each one. Representative sequences were aligned using PyNAST against Greengenes template alignment (gg_13_8 release), and a taxonomical assignment step was performed using the basic local alignment search tool (BLAST) to map each representative sequence against a combined database encompassing the Greengenes and PATRIC databases. To correctly define species richness for the analysis of between-sample diversity, known as beta diversity, the OTU table was rarefied at 4849 sequences per sample.

### Serum and mesenteric lymph nodes cytokine levels

Serum supernatants and extracts from mesenteric lymph nodes were tested for IL-6, TNF-α and IL-10 concentrations using specific ELISAs (Peprotech, London, UK), according to the instructions of the manufacturer. The detection limit was 30 pg/mL.

### Intestinal barrier

To perform western blot analysis, protein was extracted from rat ileum using RIPA buffer following the protocol of the manufacturer (Sigma Aldrich, St. Louis, MO, USA). For this study, 20 μg of proteins were separated on a 4–12% SDS-PAGE (Invitrogen, Camarillo, CA, USA) gel and transferred to nitrocellulose. Membranes were incubated overnight with antibodies to claudin-4 (Invitrogen), occludin, zonula occludens-1 and β-defensin-1 (Antibodies-online Inc., Atlanta, GA, USA). We used appropriate secondary antibodies conjugated to IR-dyes 800CW goat anti-rabbit immunoglobulin G (IgG) and 680LT goat anti-mouse IgG (H + L) (Li-cor, Lincoln, Nebraska, USA) to visualize proteins. Proteins were then scanned using the Odyssey Imaging System (Li-cor). The expression of claudin-4, occludin, zonula occludens-1 and β-defensin-1 was quantified and normalized to β-actin using β-actin antibody (Sigma Aldrich). Oxidative damage in ileal samples was assessed using the determination of MDA formation by thiobarbiturate reaction^32^,^45^. The detection limit of this assay was 0.079 nmol/mg protein.

### Liver damage

Four μm-slices from paraffin blocks were evaluated by hematoxylin-eosin staining to study the histological changes and by Masson’s trichrome staining to assess the severity of fibrosis. A semi-quantitative score was used by a single expert pathologist to blindly classify the liver samples into: 0- normal, 1- fibrosis with thin and incomplete fibrous tracts, 2- regeneration nodules with thin complete fibrous tracts, and 3- regeneration nodules and thick and complete fibrous tracts. The degree of steatosis was evaluated in: 0- normal, 1- mild steatosis, and 2- severe steatosis. The degree of hepatic fibrosis was estimated by calculating the percentage of the area stained with picro-Sirius Red (Sirius Red F3B, Gurr-BDH Lab Supplies, Poole, England). The positive stained area was quantified using a morphometric analysis system. Briefly, twelve images were obtained with an optic microscope (Nikon Eclipse E600, Nikon Corporation, Japan) at magnification of x20. Images were imported using an image-analysis system (AnalySIS, Soft-Imaging System, Munster, Germany) software and automatically merged. The positive area was the sum of the area of all positive pixels.

### Statistical analysis

Statistical analysis was performed using the SPSS statistical package (SPSS Inc. version 17.0, Chicago, Illinois, USA). All parameters are reported as percentages and mean ± SEM. Differences between groups were analyzed using the Fisher’s exact test for qualitative variables. The Shapiro-Wilk test was used to check the normality of data distribution. The Student’s t-test was used when data were normally distributed; otherwise, the non-parametric Mann-Whitney U-test was used. Correlations were calculated using Pearson’s test or Spearman’s test. The probability of developing ascites was assessed by Kaplan-Meier test and then compared using the log rank test. A two-tailed *P* value below 0.05 was considered statistically significant.

Specifically regarding the microbiome evaluation, statistical analyses were carried out in QIIME and in R. To work with normalized data, we analyzed an equal number of sequences from all groups. The Kruskal-Wallis one-way test of variance was used to compare the mean number of sequences of the groups. The analysis provided false discovery rate (FDR)-corrected *P*-values. FDR < 0.1 was considered significant for all tests.

Sample size was calculated according to previous data from our group^15^. Considering an α error of 0.05, a β error of 0.20, an expected percentage of bacterial translocation of 62% in the water group and of 8% in the probiotic group, and a 43% overall mortality during the study, we calculated that the minimal number of rats required in each group was 19.

## Additional Information

**How to cite this article**: Sánchez, E. *et al*. Fermented milk containing *Lactobacillus paracasei* subsp. *paracasei* CNCM I-1518 reduces bacterial translocation in rats treated with carbon tetrachloride. *Sci. Rep.* **7**, 45712; doi: 10.1038/srep45712 (2017).

**Publisher's note:** Springer Nature remains neutral with regard to jurisdictional claims in published maps and institutional affiliations.

## Supplementary Material

Supplementary Information

## Figures and Tables

**Table 1:** Bacteria isolated in microbiological culture from samples of homogenizate of mesenteric lymph nodes, ascitic fluid, pleural fluid, liver and spleen in cirrhotic rats treated with water or probiotic.

Group	Rat	Isolated bacteria	Laparotomy samples				
			Mesenteric lymph nodes	Ascitic fluid	Pleural fluid	Liver	Spleen
Water group	1	*E. coli*	+	−	−	−	+
	2	*E. coliEnterococcus* spp	−	−	−	+	−
	3	*E. coliEnterococcus* spp.	+	−	+	−	−
	4	*E. coli*	+	−	−	−	−
	5	*E. coli*	+	−	−	−	+
	6	*E. coliEnterococcus* spp.	+	+	−	−	−
	7	*E. coli*	+	−	−	−	−
Probiotic group	1	*E. coliEnterococcus* spp.	+	−	−	−	+

^+^represents presence of bacteria in microbiological culture, and − represents absence of bacteria in microbiological culture*. E. coli, Escherichia coli*.
