# Supplementary material for: Fermented milk containing Lactobacillus paracasei subsp. paracasei CNCM I-1518 reduces bacterial translocation in rats treated with carbon tetrachloride
Source: Sci Rep. 2017 Apr 3;7:45712. doi: 10.1038/srep45712 (PMC5377325; doi:10.1038/srep45712)
Supplement: Supplementary Information [file srep45712-s1.pdf]

Title

Fermented milk containing *Lactobacillus paracasei* subsp. *paracasei* CNCM I-1518 reduces bacterial translocation in rats treated with carbon tetrachloride

Authors

Elisabet Sánchez, Juan C. Nieto, Silvia Vidal, Alba Santiago, Xavier Martinez, Francesc J. Sancho, Pau Sancho-Bru, Beatriz Mirelis, Helena Corominola, Candido Juárez, Chaysavanh Manichanh, Carlos Guarner and German Soriano

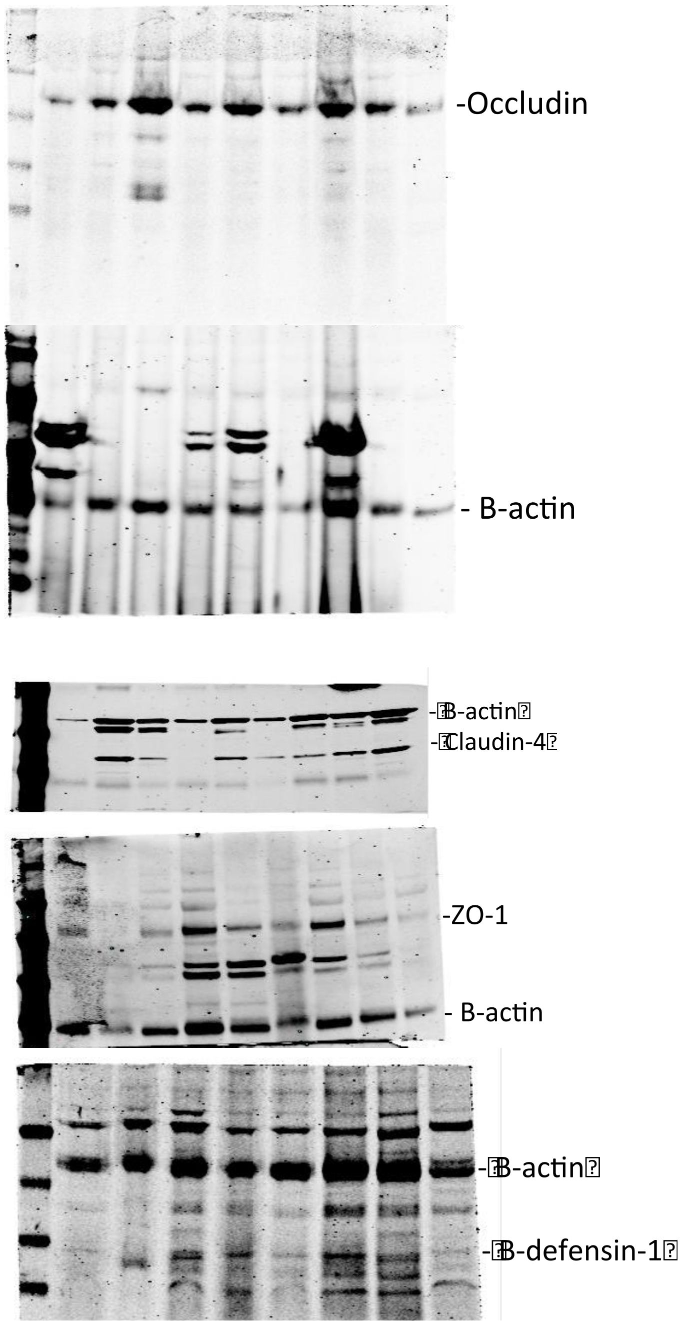

Supplementary Figure 1. Western blot membranes of intestinal barrier proteins.
20 μg of proteins were separated on a 4-12% SDS PAGE gel and transferred to nitrocellulose. Membranes were incubated overnight with antibodies to claudin-4, occludin, zonula occludens-1 (ZO-1) and β-defensin-1. We used appropriate secondary antibodies conjugated to IR-dyes 800CW goat anti-rabbit immunoglobulin G (IgG) and 680LT goat anti-mouse IgG (H+L) (Licor) to visualize proteins. Proteins were then scanned using the Odyssey Imaging System (Li-cor).
